# Antigen-presenting cell internalization is key for understanding and evaluating therapeutic antibodies’ immunogenicity

**DOI:** 10.3389/fimmu.2025.1617395

**Published:** 2025-09-05

**Authors:** Maria Lteif, Marc Pallardy, Isabelle Turbica

**Affiliations:** Université Paris-Saclay, INSERM, Inflammation, Microbiome and Immunosurveillance, Orsay, France

**Keywords:** therapeutic antibodies, dendritic cells, internalization, immunogenicity, immunoglobulin

## Abstract

Therapeutic antibodies have revolutionized the treatment of many diseases. However, their safety and efficacy are often altered by their immunogenicity, as many patients frequently develop anti-drug antibodies. Dendritic cells (DCs) are the most potent antigen-presenting cells of the immune system. DCs initiate the immunogenic adaptive immune response by internalizing therapeutic antibodies using different pathways and receptors, leading to antigen presentation to T-cells. Recently, studies have shown that the uptake of antibodies by immune cells could contribute to their immunogenicity. This review will present in detail the different DC internalization mechanisms and then discuss the impact of therapeutic antibodies’ properties and aggregation on their uptake by DCs and, therefore, their immunogenicity. We will also highlight cellular models and strategies used to evaluate antibodies’ internalization. Addressing the uptake of antibodies by DCs could help to predict the risk of immunogenicity and to develop mitigation strategies.

## 1 Introduction

Therapeutic antibodies (Abs) have emerged as the fastest-growing class of biotherapeutics and are used to treat and detect many diseases (1). However, they can elicit unwanted immunogenicity in a subset of patients, leading to the production of anti-drug Abs (ADAs). This implicates the development of a T-cell-dependent immune response. This response is initiated by the internalization of Abs by antigen-presenting cells (APCs), such as dendritic cells (DCs), followed by the processing of these Abs into peptides for presentation on major histocompatibility (MHC) class II molecules on the cell surface. These peptides are recognized by cognate T cells, resulting in their activation, which in turn activates antigen-specific B cells that mature into Ab-secreting plasma cells (2). Recently, measurements of Ab internalization in DCs have been proposed as a tool for immunogenicity risk assessment (3–6). These assays have shown that the rate of Ab internalization can influence immunogenicity risk (4, 6–8). However, different factors contribute to ADA generation, including the intrinsic properties of the therapeutic protein, the therapeutic regimen, and the patient-specific characteristics such as its immune status or genetic background (9).

Understanding the factors influencing Ab internalization into DCs could help optimizing their design and reducing their immunogenic risk. This review aims to explore the structure and function of Abs, the mechanisms of Abs’ internalization by DCs, and the impact of Abs’ properties on this process. By examining these aspects, we will highlight how these factors influence the immune response and discuss the current methods for evaluating Abs’ internalization *in vitro*. Finally, we will propose an evidence-based strategy for evaluating the immunogenicity risk of therapeutic Abs candidates, including internalization assessment.

Immunoglobulin (Ig)-based therapeutic Abs have dominated the biotherapeutic field mainly due to their structural and functional properties. However, alternative Abs formats have been developed over the past two decades. These include smaller or engineered constructs designed to improve biodistribution, enhance specificity, or reduce side effects. Importantly, the current preclinical and clinical landscape now includes a wide array of structurally and functionally diverse Ab-based molecules such as single-domain Abs/nanobodies, multispecific Abs (e.g., bispecific, trispecifics), polymeric Abs such as IgA dimers and IgM pentamers, Ab-drug conjugates) (10). Numerous novel Abs are currently being investigated in clinical trials, and the number of monoclonal Abs receiving marketing authorization continues to grow. This evolving landscape is reviewed annually in the “Antibodies to Watch” series (1).

Abs or Igs mediate the humoral response and are most effective against extracellular pathogens (11). They are typically composed of light chains (LCs) and heavy chains (HCs). There are two LC isotypes, kappa (κ) and lambda (λ), and five HC isotypes, gamma (γ), alpha (α), mu (µ), delta (δ), and epsilon (ϵ) which define the Ab isotype (IgG: γ, IgA: α, IgM: µ, IgD: δ, IgE: ϵ) and confer distinct effector functions (12, 13). Importantly, the overall structure and composition of Ig classes differ significantly. While IgG, IgA (monomeric), IgD, and IgE exist as monomers composed of two LCs and two HCs, IgM typically forms a pentameric structure (and occasionally a hexamer), comprising five Ig units linked by disulfide bonds and a joining (J) chain. This results in a molecule containing 10 HC and LC and confers high valency and strong avidity for antigens. Dimeric forms of IgA also include a J chain and are predominant in mucosal secretions (12, 14). Both HC and LC are composed of constant and variable domains. The amino-terminal variable regions of the HC and LC (VH and VL, respectively) together form the antigen-binding site, shaped by three hypervariable loops known as complementarity-determining regions (CDRs 1, 2, and 3), which are separated by conserved beta-sheet framework regions (FRs). The constant region determines the Ab’s effector functions and varies by isotype. The γ, α, and δ heavy chains contain three constant domains (CH1, CH2, and CH3) and a hinge region, which provides flexibility and enhances antigen binding and cross-linking. In contrast, the µ and ϵ heavy chains include a fourth constant domain (CH4), which replaces the hinge region and contributes to a more rigid structure (15).

Based on enzymatic cleavage, the Ab can be divided into functionally distinct regions: two fragment antigen-binding (Fab) regions and one fragment crystallizable (Fc) region. The Fab region contains the VH, VL, CH1, and CL domains and mediates antigen recognition, while the Fc region—comprising CH2 and CH3 domains (and CH4 in IgM and IgE)—is responsible for interacting with immune effector molecules (16). These functions are determined by their interactions with different binding partners: antigens or therapeutic targets through their Fab region, Fc receptors (FcR) for IgG (FcγRs), the neonatal Fc receptor (FcRn), and the complement system via their Fc portion (17). The main mechanisms of action of Abs include antagonism or agonism for a soluble ligand or receptor and blockade of cell–cell interaction. Ab-receptor or target binding at the cell surface can thus lead to the endocytosis of the formed complex (18, 19). On the other hand, the Fc-mediated effector functions comprise Ab-dependent cell-mediated cytotoxicity (ADCC), Ab-dependent cellular phagocytosis (ADCP), and complement-dependent cytotoxicity (CDC) (20). NK cells and macrophages express FcγRIIIa (CD16a) on their surface, recognizing Abs’ Fc portion and are the major mediators of ADCC, resulting in target cell killing. This mechanism plays a key role in the mode of action of therapeutic monoclonal Abs such as rituximab (anti-CD20) and cetuximab (anti-EGFR) in immunotherapy (21).

## 2 Mechanisms of internalization by dendritic cells

The mechanisms of therapeutic Ab internalization by DCs depend on the physico-chemical properties of the Abs, which influence the rate of uptake and the subsequent immune responses. This internalization process is also significantly affected by the expression levels of the target antigens on the DC surface. Higher antigen density typically facilitates more efficient Ab binding and uptake, thereby enhancing antigen processing and presentation, critical steps for initiating effective immune responses. Recent studies suggested a link between mAb’s internalization rate and their immunogenicity (4, 22). Before considering the relevance of different endocytic pathways for Abs by APCs, and particularly DCs, we will first summarize the internalization pathways utilized by DCs.

**Figure 1:**
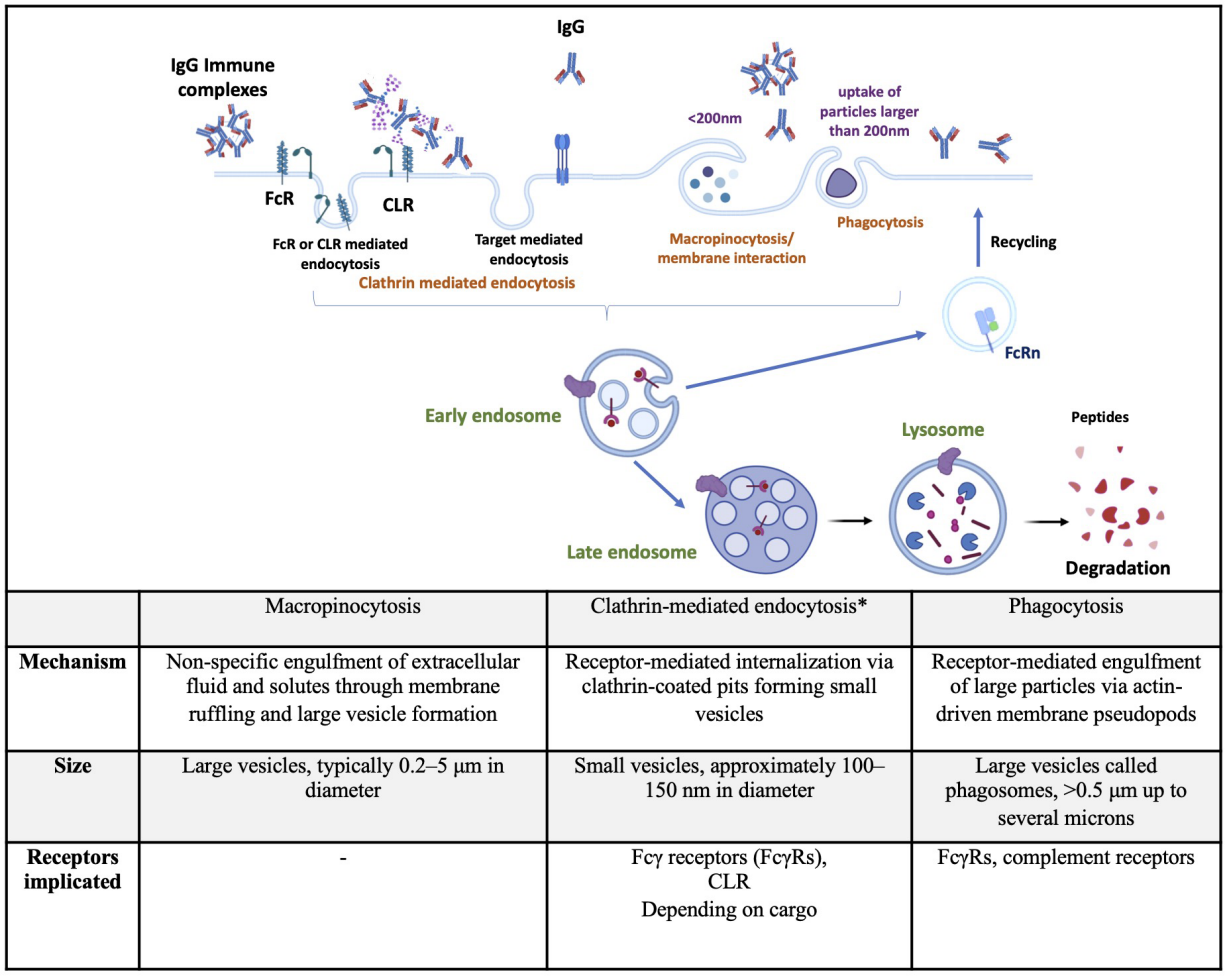
Summary of endocytic pathways implicated in therapeutic antibodies internalization by dendritic cells. Top: Schematic representation of major endocytic routes used by dendritic cells to internalize IgG or immune complexes, including clathrin-mediated endocytosis, macropinocytosis, and phagocytosis. Bottom: Tabular summary comparing the key features of each pathway, including the mechanism of internalization, vesicle size, and receptors involved. *includes target-mediated endocytosis. CLR, C-type lectine receptor; FcR, Fc receptor; FcRn, neonatal Fc receptor;IgG, immunoglobulin G. Created using BioRender.com.

Internalization pathways include macropinocytosis, clathrin-mediated endocytosis (CME), clathrin-independent dynamin-dependent endocytosis or fast endophilin-mediated endocytosis (FEME), clathrin-independent carrier/glycosylphosphatidylinositol-anchored protein-enriched early endocytic compartment (CLIC/GEEC), phagocytosis, and caveolae-dependent endocytosis. These pathways have been described in detail in a previous review (3). Macropinocytosis, CME, and phagocytosis, the three main mechanisms implicated in DC internalization of therapeutic Abs, are described below and summarized in **Figure 1**. The FEME, CLIC/GEEC, and caveolae-dependent pathways are less described in the literature as involved in Abs uptake. Studies suggest their role in oxidized low-density lipoprotein (23), polyomavirus particles (24), and Simian Virus 40 uptake (23) by immune cells.

Macropinocytosis occurs constitutively in DCs and mediates the non-specific uptake of soluble antigens. It is initiated by the polymerization of actin at the plasma membrane, leading to the formation of ruffles and large endocytic vesicles known as macropinosomes (25). This process is regulated by the small GTPases Rac1 and Cdc42, involved in actin polymerization, (26) modifications of the submembranous pH, and the activity of Na+/H+ exchangers (27). Macropinosomes destined for antigen presentation undergo a defined sequence of maturation steps involving acidification, fusion with endosomal compartments, and enzymatic degradation to process their contents, a series of events detailed later. Briefly, they might recycle back to the plasma membrane or undergo a series of changes that ultimately lead to their interaction and fusion with endolysosomal compartments, where their cargoes are degraded by hydrolytic enzymes (28). Since DCs are specialized in antigen capture, considerable research effort has been expanded to study the mechanisms of antigen uptake in these cells. Many of these studies use pharmacological inhibitors to study macropinocytosis. However, these inhibitors are notoriously non-specific, which limits the understanding of the mechanisms underlying constitutive macropinocytosis (29). For instance, amiloride derivatives inhibit macropinocytosis by impairing Na+/H+ exchanger activity, which is important for actin remodeling mediated by Rho family GTPases (30). Similarly, wortmannin, which inhibits PI3 kinase activity, also suppresses actin polymerization, a process involved in other internalization mechanisms, making the interpretation of these results more complex (25, 31).

CME is the most studied and well-characterized endocytic mechanism, as it constitutes the major endocytic pathway in mammalian cells. This pathway is essential for regulating cell signaling and mediates nutrient uptake (32). Upon binding, activated receptors are phosphorylated by G protein-receptor kinases, which in turn recruit adaptor proteins and initiate a cascade leading to the formation of clathrin-coated pits. These pits rapidly invaginate and pinch off to form clathrin-coated vesicles, then outline pathways of uncoating, transport, and fusion with early endosomes to deliver their cargo (33, 34). DCs express several receptors mediating CME, such as C-type lectin receptors (CLR), sialic acid binding immunoglobulin (Ig)-like lectins (Siglecs), death receptors (Fas, TNF, and TRAIL receptors), scavenger receptors (CD36, SR-A), and FcR (35–37). In this review, we will mainly focus on CLR and FcR, as the literature indicates that these are the most implicated receptors in the cellular uptake of therapeutic Abs. Siglecs recognize sialic acid via their extracellular domain (38). Scavenger receptors bind and internalize modified low-density lipoproteins, and death receptors and damage-associated molecular patterns that are released during different cell death mechanisms will not be further detailed in this review, as they are not implicated in Abs uptake. CLRs are a diverse family of soluble and transmembrane proteins that bind carbohydrates in a calcium-dependent manner using a conserved carbohydrate recognition domain (CRD) (39). Type I transmembrane CLRs possess various CRD and comprise the mannose receptor family, such as DEC-205 (CD205 or LY75) and the macrophage-mannose receptor (MMR or CD206), both implicated in antigen uptake. Type II transmembrane CLRs have a single CRD domain and include DC-associated C-type lectin 1 (Dectin-1 or CLCE7A), Dectin-2 (CLEC6A), macrophage-inducible C-type lectin (Mincle or CLEC4A), the dendritic-cell-specific ICAM3-grabbing nonintegrin (DC-SIGN or CD209), and DC NK lectin group receptor-1 (DNGR-1 or CLEC9A). These receptors are involved in pathogen recognition and the shaping of innate immune responses (40). MMR (CD206) and DC-SIGN (CD209) are some of the major mannose-binding CLRs in human DCs (41, 42). CD206 and CD205 recognize glycans that terminate in mannose, fucose, or N-acetylglucosamine (43, 44). Whereas these two receptors bind preferentially to a single residue, CD209 also binds terminal high-mannose (HM) glycans (45). Ig’s glycosylation (N-and O-glycosylation) is one of the major co‐translational modifications and/or post‐translational modifications (PTMs) (46). FcRs that recognize the Fc part of Abs are classified according to the Ig isotype: FcγR and neonatal FcRn bind IgG, FcαR binds IgA, FcϵR binds IgE, FcμR binds IgM, and FcδR binds IgD (47). Since IgG are the main used isotype among Abs in the therapeutic field (48), we will mainly focus on FcγR and FcRn. FcγRs are broadly classified as activating or inhibitory, depending on the signaling properties of their intracellular domain. In humans, activating FcγRs include FcγRI, FcγRIIa, FcγRIIc, and FcγRIIIa, signaling through immunoreceptor tyrosine activating motifs (ITAMs). FcγRIIb represents the only inhibitory FcγR, signaling through an immunoreceptor tyrosine inhibitory motif (ITIM) in its cytoplasmic region (49). *In vitro*, APC expresses a wide range of FcγRs with a potential role in antigen presentation. Human monocyte-derived DCs (moDCs)s express mainly the activating FcγRIIa and the inhibitory FcγRIIb receptors, and to a lesser extent, FcγRI and FcγRIIIa. Human *in vitro* generated macrophages express all FcγRs with high levels of FcγRIIa (50). FcγRs can also be classified by their binding affinities to human IgG: FcγRI binds monomeric IgG with high affinity, whereas FcγRII and FcγRIII bind multimeric IgG or immune complexes (ICs) with very high affinity (51), and also Abs aggregates. Binding of IgGs to activating FcγR leads to ITAM phosphorylation, activation of the Src-Syk pathway, FγR-IgG ICs’ internalization, and routing to lysosomes. Syk activates downstream signaling molecules, primarily increasing calcium flux and the activation of protein kinase C (52) These intracellular changes lead to activation of Rho GTPases and actin remodeling, which is critical for IC’s internalization (53). The studies on therapeutic Abs internalization through the FcRs are detailed in the next section.

The FcRn binds to the Fc portion at acidic pH in the early endosome and recycles IgGs to the plasma membrane to be released at neutral pH (54), rescuing them from lysosomal degradation and extending their serum half-life (55). At higher concentrations of IgG, FcRn becomes saturated, leading to a smaller proportion of IgG being routed to the lysosomal compartments. The latter trafficking also occurs when FcRn binds to multimeric IgG, such as aggregates or ICs (54). Importantly, FcRn saturation also affects the processing of Abs in DCs’ endolysosomal compartments (56). If the FcRn-mediated IgG recycling is saturated, a greater proportion of IgG molecules is directed to lysosomal degradation within DCs (54, 57). This enhances antigen presentation and may increase immunogenicity by promoting T-cell activation against the therapeutic Ab. This mechanism highlights a key consideration in clinical Ab therapy, where FcRn saturation can influence both pharmacokinetics and immune responses.

Phagocytosis consists of the recognition of particles larger than 0.5µm in diameter by phagocytic receptors and their uptake into a plasma membrane-derived vesicle, known as phagosomes. In humans, phagocytosis is restricted to specialized cells called phagocytes, including macrophages, neutrophils, and DCs (58). Plasma membrane receptors of phagocytes are divided into non-opsonic or opsonic receptors. Non-opsonic receptors recognize directly distinct molecular patterns on the particle and include C-type lectins, such as Dectin-1, Dectin-2, Mincle, or DC-SIGN. Opsonic phagocytic receptors include FcRs and complement receptors recognizing Ab- or complement-opsonized particles, respectively (59). C3bi is the major opsonin recognized by the complement receptor on APCs, thus leading to the opsonized particle’s phagocytosis (60). After ligand binding, phagocytic receptors initiate signaling pathways leading to modification in the membrane composition and regulation of the actin cytoskeleton, therefore resulting in the formation of pseudopods covering the particle. Additional pseudopods are generated around the target, forming a phagocytic cup that closes up at their distal margins to form phagosomes (61).

Phagocytosis is relevant to the immunogenicity of therapeutic Abs because it governs the uptake, processing, and presentation of Ab-derived peptides on MHC molecules, which in turn can activate T cells and start adaptive immune responses. Evidence supporting this comes from studies demonstrating that FcγR-mediated phagocytosis enhances antigen processing and cross-presentation by DCs, promoting robust CD4+ and CD8+ T cell responses (49). Furthermore, alterations in the Fc glycosylation pattern of Abs can modulate their interaction with FcγR and phagocytic uptake, thereby influencing their immunogenic potential (62).

Importantly, this process is also shaped by factors such as the size of the Ab or ICs, as well as the binding affinity of phagocytic receptors—topics that will be explored in the following section. Collectively, these observations highlight phagocytosis as a critical determinant in shaping the immunogenicity profile of therapeutic Abs.

## 3 Fate of internalized peptides

As for other proteins, there is strong evidence that the trafficking fate of internalized Abs is common for all major endocytosis pathways. Internalized protein particles are routed to the early endosomes, also called the sorting endosomes (63). In this sorting station, internalized material is either recycled back to the plasma membrane or routed from the early endosome to late endosomes and lysosomes for degradation for a subsequent presentation (64). Early endosomes are characterized by the presence of the GTPase Rab5, which regulates endocytic membrane trafficking by recruiting several effectors. They will become increasingly acidic as endosomes mature from early/recycling to late endosomes. This process requires the switch from Rab5 to Rab7 to drive the maturation of early endosomes into late endosomes, which can then fuse with lysosomes for cargo degradation (65). In the lysosomes, cathepsins are the most abundant proteases responsible for degrading macromolecules. They comprise 12 members, mainly endopeptidase-cleaving peptide bonds. Among them, cathepsin S, a cysteine protease, has a central role in degrading antigens in APCs and their presentation in association with MHC-II molecules to CD4+ T cells (66). In fact, MHC-II molecules bind peptides of around 13 to 25 amino acids generated by this proteolysis process (67). Efficient peptide binding requires the access of MHC-II molecules to lysosomal compartments. Newly synthesized MHC class II molecules associate with the invariant chain (Ii), preventing premature ligand binding in the ER and directing the complexes to the endocytic compartment (68). Ii is proteolyzed, and the resulting CLIP occupies the peptide-binding groove (69). HLA-DM facilitates the release of CLIP and stabilizes the empty MHC class II until an exogenous peptide is loaded. The MHC class II-peptide complex is then transported to the cell surface for recognition by CD4+ T cells (70). Activation of antigen-specific CD4+ Th cells helps activate cognate antigen-specific B cells to proliferate and differentiate into plasma cells producing high-affinity ADA (71).

## 4 Impact of antibodies properties on their internalization

The internalization of therapeutic Abs by APCs is influenced by their properties, such as size, post-translational modifications, charge distribution, hydrophobicity, degradation, and aggregation.

### 4.1 Size

The size of internalized protein particles mainly impacts the route of internalization. While small particles tend to be internalized via fluid-phase endocytosis, bigger complexes and aggregated peptides or proteins are probably uptaken by phagocytosis (72). These uptake pathways differ in how they deliver antigens to intracellular processing compartments, potentially influencing the efficiency and nature of antigen presentation to the immune system (73, 74). FcγRs generally exhibit low affinity for monomeric IgG, preferentially binding ICs that enable receptor cross-linking and subsequent internalization. Although FcγRI (CD64) can bind monomeric IgG with higher affinity and mediate Ab internalization *in vitro* (e.g., for avelumab (75)), most FcγRs require multivalent ICs or antigen bound IgGs to initiate uptake and signaling (15, 76). Endogenous IgGs are present at high concentrations in plasma (~10 mg/mL) (77), exceeding therapeutic Ab levels, leading to FcγRI saturation under physiological conditions (78). This saturation limits FcγR availability for free therapeutic Ab, which compete with endogenous IgGs for receptor binding (76). When Abs’ targets are soluble and circulate in the bloodstream, IC formation facilitates effective FcγR engagement (79), thereby influencing Ab clearance and immunogenicity. This competitive and dynamic interplay underscores that IC formation, rather than free Ab concentration alone, primarily governs FcγR-mediated uptake. Multimeric Abs and large ICs are primarily routed to lysosomes as they escape FcRn binding, resulting in their degradation into peptides that are later loaded onto MHC class II molecules (54). This increases their potential to trigger CD4^+^ T cell responses, a key step in the development of ADA. On the other hand, stress occurring during handling or administration can favor aggregation (80). Aggregated forms of Abs more readily uptaken by immune cells (81–83), as they engage low-affinity FcR more effectively than monomeric Abs (49). This enhanced engagement may promote immune recognition by facilitating antigen processing and presentation. Thus, both the size and aggregation state of therapeutic Abs can significantly influence their internalization route, intracellular fate, and ultimately, their immunogenicity risk.

### 4.2 Protein modifications and their impact on recognition by specific receptors

**Figure 2:**
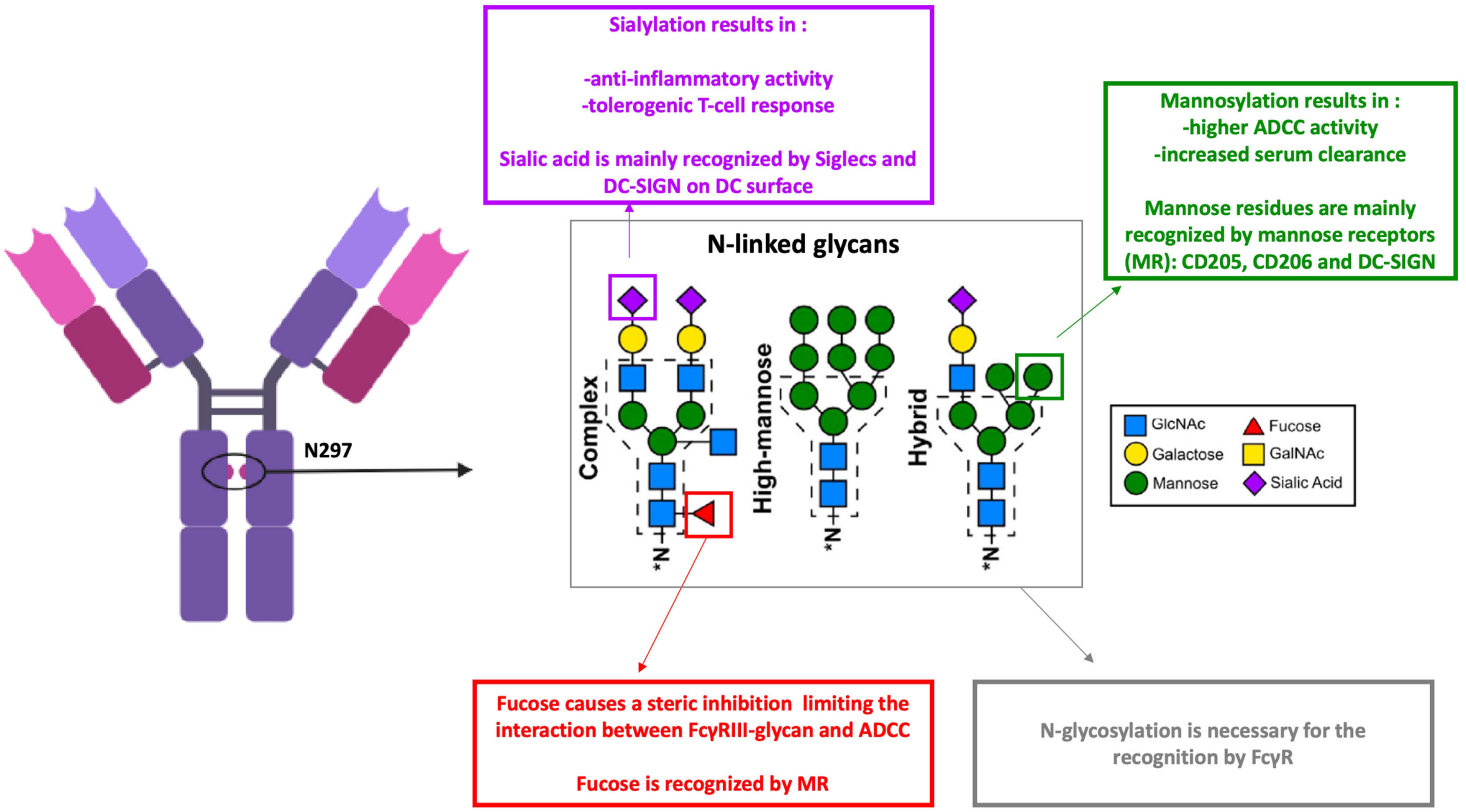
Antibodies N-glycans and their recognition by dendritic cells receptors. ADCC, antibody dependent cell-mediated cytotoxicity; FcγR, Fc gamma receptors; MR, mannose receptors. Monoclonal antibodies (mAbs) glycosylation modifies their functional properties and impacts their recognition by dendritic cell (DC) receptors through carbohydrate residues. Sialic acid is primarily recognized by Siglecs and DC-SIGN on the surface of DCs. In contrast, fucosylation of mAbs reduces their interaction with FcγRIII, leading to decreased antibody-dependent cellular cytotoxicity (ADCC). However, mannosylation of mAbs enhances ADCC activity and is associated with increased clearance. Mannose-sensitive receptors, such as CD205, CD206, and DC-SIGN, are capable of recognizing fucose and mannose residues. Created using BioRender.com.

Glycosylation is a common post-translational modification that occurs during the production of Abs. A significant proportion of endogenous IgG Abs naturally exhibit N-glycosylated residues in their Fab regions (84, 85). However, Fab glycosylation is generally avoided in therapeutic Abs primarily for stability and manufacturability reasons. Despite this, certain studies have highlighted potential benefits of Fab glycosylation under specific contexts. For example, Fab sialylation has been shown to improve the serum half-life of Abs, such as cetuximab (an anti-EGFR monoclonal Ab (86). Courtois et al. showed that introducing glycans to shield aggregation-prone regions enhances Ab’s stability to a comparable extent as replacing hydrophobic amino acid residues with hydrophilic ones (87). Knowledge of the biological role of Fab-associated glycans in immunity remains limited, necessitating further investigation to elucidate their functional significance. On the other hand, IgGs contain a conserved N-glycosylation site at the asparagine 297 (Asn297 or N297) residue within their Fc region, which modulates their interactions with CLR and FcγRs. The type of oligosaccharides found in the conserved Asn297 glycosylation site of Ab Fc portions depends on the mode of production. Mammalian systems generally result in complex-type biantennary oligosaccharides in the Fc portion (62). The oligosaccharides in the Fc region comprise HM glycan, containing five to nine mannose residues that are recognized by mannose receptors (MRs), complex glycans in which “antennae” initiated by N-acetyl-d-glucosamine (GlcNAc) extend the core, and hybrid glycans, which are the combination of HM and complex glycans (88) (**Figure 2**). For biantennary N-glycans, additional fucosylation, galactosylation, and sialylation may occur (89). APCs expressing many CLR can recognize the glycans entities on therapeutic Abs. While endogenous human IgG1 contains relatively low percentages (0.1%) of HM glycans, recombinant IgG might contain up to 10% depending on the producing cell line (90). Studies have shown that HM has potentially advantageous biological activities. Zhou et al. demonstrated that mannose resulted in a higher ADCC activity and an increased binding affinity to FcγRIIIa (91). These properties could be explained by the lack of core fucose in addition to the presence of mannose-ending glycans (92). Similar results were found by Yu and collaborators but they demonstrated a negative impact on CDC (93). On the other hand, HM residues influence Ig’s pharmacokinetics with an increased serum clearance of oligomannose glycoforms, which are recognized by mannose receptors and rapidly eliminated from circulation (94). Fc’s HM glycans are recognized by DC-SIGN and MR,and serum mannose-binding lectin (95). These mannose-sensitive receptors are shown to be implicated in the internalization of viruses (96) and factor VIII procoagulant protein (FVIII) (97). Moreover, internalization of mannosylated antigens are associated with an enhanced uptake by DC and therefore with an enhanced presentation to CD4 T cells (97, 98). Few studies focused on therapeutic Abs’ uptake through mannose-sensitive receptors. Wolf et al. showed an increased internalization of mannosylated rituximab compared to the wild-type form which colocalized in the lysosome (99). Interestingly when therapeutic Abs are injected SC, the subcutaneous compartment lacks the serum alpha-mannosidase that trims the substrate Man9, and therefore, M9–6 glycans are not converted into M5 types and may interact with cutaneous DC through DC-SIGN (100). However, recognition by these specific CLRs can shape T-cell responses. For instance, mannosylated antigens internalized via the mannose receptor (MMR) enhance antigen presentation and T-cell activation (reviewed in (101)). This was also observed with mannosylated rituximab, which led to increased T-cell activation in 50% of tested donors compared to the wild-type Ab. Similar findings have been reported for antigens engaging DC-SIGN (102). Wawrzyniak et al. found that fucosylation of adalimumab does not influence its uptake by moDCs nor their activation (103). Human IgGs contain low levels of sialylated Fc (5%–10%). Studies have shown that sialylation of the N-linked glycan conveys Abs an anti-inflammatory activity. The dependency of this effect on the interaction between DC-SIGN and sialylated Fc remains under debate (104). Moreover, the uptake of sialic acid-modified antigens by DCs results in the initiation of a tolerogenic T-cell response (105). Wolf et al. showed that the hypersialylated variant of rituximab had a decreased internalization rate by DC and weak or nearly no co-localization in lysosomal compartments (99). Besides their recognition by CLR, Fc fragment glycosylation on Abs is also crucial for its interaction with FcR (106). The presence of fucose on IgG N-linked glycan is a modification of the human IgG1 Fc structure with many functional consequences. The core fucose causes a steric inhibition, limiting the interaction between FcγRIII-glycan and IgG-Fc and, therefore, leading to a suboptimal affinity (107). Hence, afucosylated IgG bind to FcγRIIIa and FcγRIIIb with a higher affinity (108). Studies have shown that a fucosylated IgG induces FcγRIIIa-dependent signaling and promotes ADCC (109, 110), the major mode of action to deplete tumor cells. Macrophages also express FcγRIII, which could mediate phagocytosis of opsonized IgG. However, many studies report FcγRI as the major implicated receptor (111, 112). Afucosylated therapeutic Abs are not generally reported to enhance phagocytosis significantly. On the other hand, Abs can be engineered to reduce or completely eliminate their interaction with FcγRs, thereby minimizing their effector functions such as ADCC and CDC. This approach is particularly applied to Abs used in oncology to reduce off-target toxicity and enhance therapeutic efficacy. One common strategy involves removing the N-linked glycosylation site at Asn297 as exemplified by atezolizumab, an anti-PDL1 (113), or introducing silencing mutations such as the so-called LALA mutation (Leu234Ala together with Leu235Ala) to reduce or abolish FcγR binding and effector functions like ADCC/CDC (114). Glycan residues on the Fc region of IgG critically influence the recruitment and binding affinity of FcγR on DCs, thereby modulating Ab internalization and directing their intracellular trafficking and immunological fate (78, 115). Jin et al. showed a higher internalization level of avelumab through FcγRI-mediated internalization in comparison to the non-glycosylated variant (75). The Fc-mediated internalization of ICs is associated with enhanced antigen uptake and presentation by DCs and macrophages (50, 116, 117). Amigorena et al. showed the dependency of this enhanced antigen presentation on tyrosine residues of the cytoplasmic domain of ITAM as the mutation of these residues motif inhibited both the internalization of IgG–antigen complexes and the presentation of the IgG-coated antigen (118). FcγR activation by ICs also induces their sequestration in intracellular vesicles for lysosomal degradation and antigen processing (119). However, beyond the internalization step, FcγRs vary in their intercellular trafficking capacities of ICs. ICs transported by the full-length FcγRIA are routed to MHC-II compartments, whereas those up taken by a truncated, tail-deleted FcγRIA are redirected to recycling compartments, resulting in reduced antigen presentation (120). Thus, the uptake pathway might significantly influence their functional response. FcγRIIb also plays a role in antigen capture. However, its inhibitory role is controversial; some studies have shown that FcγRIIb deficiency results in an enhanced potential to generate antigen-specific T cell responses, while others indicate that FcγRIIb-mediated uptake by DCs can elicit a weak T cell response (121, 122). Since both glycan recognition by CLR and FcR binding modulate immune cell activation and antigen presentation, these molecular modifications may play a crucial role in clinical immunogenicity by influencing the likelihood of immune responses. However, translating *in vitro* findings on FcγR-mediated internalization to clinical settings is challenging, and many aspects should be considered, such as the competitive receptor occupancy by endogenous IgGs, the dynamic formation of ICs *in vivo* and the receptor expression profiles on relevant cell types. The use of humanized Abs may therefore provide more predictive insights into FcγR interactions and therapeutic Ab fate in patients (76, 123).

Another modification that could influence therapeutic Abs receptor recognition is PEGylation. Polyethylene glycol (PEG) is a flexible, hydrophilic polymer that is chemically attached to therapeutic Abs after the purification process. PEG enhances the half-life of Abs by creating a steric barrier that reduces protein binding, thereby decreasing the frequency of administration. This technique, known as PEGylation, can resemble to post-translational modifications of proteins and is clinically proven as an effective strategy for extending the half-life of protein-based therapies (124). Sanchez and collaborators showed that partial coating of PEG reduces the macrophage uptake of particles (125). Other studies demonstrated a reduced internalization of nanocarriers with a longer PEG chain by macrophages (126, 127). Recently, de Bourayne et al. showed that pegylation of certolizumab reduces its uptake by DC, peptide presentation to T-cells, and T-cell priming (128). PEGylation is frequently associated with diminished immunogenicity by masking immune epitopes (129, 130). PEGylation shields therapeutic Abs from recognition and internalization by immune cells, thereby playing a key role in minimizing clinical immunogenicity.

### 4.3 Charge distribution and hydrophobicity

Abs’ behavior is influenced by their surface characteristics. Results have demonstrated that modifying the charged and hydrophobic regions can enhance the solution properties of Abs (131, 132). However, positive charge patches were also described as adversely affecting Abs pharmacokinetic (133). The presence of large positively charged patches in the CDR can lead to non specific binding (134). On the other hand, hydrophobicity as well as surface charges have been linked to undesirable aggregation in IgG (135, 136). Studies have shown a higher internalization rate of positively charged polymeric nanoparticles following an ionic interaction with negatively charged membranes (137, 138). Jin et al. observed *in vitro* a lower internalization of a variant of avelumab (anti-PD-L1) with lower isoelectric point (75). Moreover, bococizumab, an anti-PCSK9 Ab with excess positive charges, showed poor pharmacokinetics properties and high immunogenicity (139). Wen et al. showed its high internalization rate *in vitro* by moDCs in comparison to other Abs (8). Liu et al. also found that positively charged Abs exhibited enhanced cellular uptake (140). Thompson et al. observed an accumulation of transferrin and an endosomal maturation following positively charged proteins’ internalization, thus suggesting an alteration of peptide processing and presentation (141). Another recent study also showed that adding positive residues to therapeutics Abs resulted in their lysosomal accumulation and the increase of antigenic presentation (5), two critical steps of immune response initiation. Other studies showed a correlation between particle uptake and their hydrophobicity (142, 143) following an improvement of their interaction with the cellular membrane. This effect is interesting for enhancing Ab drug conjugates (ADC)s antitumor activity by favoring cell penetration (144). Together, these findings emphasize that both charge distribution and hydrophobicity can influence how therapeutic Abs are internalized, processed, and presented by immune cells.

### 4.4 Aggregation

Abs aggregation occurs during storage manufacturing, processing, storage, handling, and administration due to exposure to different stresses such as temperature variation, shaking, or light exposure (145). This stress results in Ab’s partial unfolding, leading to monomer-monomer association followed by nucleation and growth (80). Changes in hydrophobicity and charge, which are probably linked to an increased uptake, also promote aggregation (146). Even though it is now well accepted that Abs aggregates enhance immunogenicity, few data are published concerning their internalization by DCs. Ahmadi et al. showed that aggregated rituximab has a higher internalization rate compared to the native Abs, and it rapidly accumulates in late endosomes associated with antigen presentation (147). Another study identified the implication of FcγRs in ADC aggregates internalization into non-target cells compared to the control (148). Moreover, using MHC-Associated Peptide Proteomics assay (MAPPs), Rombach-Riegraf et al. identified a higher number of Ab-specific peptides associated with MHC class II molecules after loading moDCs of stir-stress aggregated Abs. According to the authors, these results suggest a higher uptake (149). The factors mentioned above impact mAb’s internalization rate and presentation by DCs. An increased presentation of internalized particles might allow the recruitment of T cells with a lower T cell receptor avidity. Moreover, modifications such aggregation can lead to structure and sequence modification, which could lead to the generation of neoepitopes priming T-cells. Therefore, evaluating Abs’ internalization should be considered part of *in vitro* testing of therapeutic Ab immunogenicity.

## 5 Target-mediated internalization

The target-mediated endocytosis is a process initiated by the binding of ligands to specific receptors on the cell surface.This leads to the formation of receptor-ligand complexes that initiate receptor-mediated endocytosis. During internalization, these complexes are internalized into early endosomes then either recycled back to the plasma membrane or degraded in lysosomes. The fate of these complexes is often determined by their pH-dependent dissociation: ligands that dissociate rapidly in acidic endosomal conditions tend to promote receptor recycling, whereas complexes that remain intact under low pH conditions are targeted for lysosomal degradation (150). Target expression by DCs facilitates therapeutic Abs’ uptake. For example, atezolizumab, binds to PD-L1 expressed on DCs as well as tumor cells, influencing immune checkpoint regulation and enhancing antitumor immunity (151). A study demonstrated that high uptake of radiolabeled anti-PD-L1 occurred in the liver and spleen, corresponding to its binding to PD-L1 receptors expressed on lymphocytes and DCs in these tissues (152). Jin et al. also showed that a variant of avelumab (an anti-PD-L1) deficient in PD-L1 binding exhibited significantly reduced internalization by immune cells, highlighting that PD-L1 engagement is critical for efficient receptor-mediated uptake of the Ab (75). Another group demonstrated that anti-TNF Abs undergo rapid target-mediated endocytosis following their binding to transmembrane TNF on the DC surface followed by its routing to degradative compartments (153). Internalization and processing of Ab-receptor complexes by DCs can potentially modulate immune responses by enhancing antigen presentation. The degradation of therapeutic Abs in lysosomes could lead to the generation of immunogenic peptides that might provoke ADA responses, impacting treatment efficacy and safety. Therefore, understanding target-mediated endocytosis is essential not only for optimizing therapeutic Ab design and delivery but also for anticipating and managing immunogenicity-related challenges in clinical applications.

## 6 Assays to evaluate internalization

As internalization gained interest in the last decade, different *in vitro* assay formats were developed (**Table 1**). Endocytosis mechanisms are commonly evaluated by flow cytometry and fluorescence microscopy (154), both requiring tracking of the molecule by direct or secondary labeling. Direct Abs labeling with a fluorescent dye is a widely used method to study their internalization (147, 155). Although this technique is straightforward, it is partially limited by the difficulty of discriminating between surface-bound and effective-internalized material and the lack of information concerning the intracellular trafficking. This could be completed by microscopic study or by adding more controls. For instance, incubating cells at 4°C inhibits the internalization, and the measured fluorescence corresponds to the surface binding signal. Washing with an acid buffer or using quenchers like trypan blue could also remove the cell surface binding signal (156). Alternative methods based on direct labeling with a pH-sensitive dye, either reactive to amine or directed to heavy chain N-linked glycans, are developed to evaluate the endocytosis and to assess lysosomal degradation. Using this technique, Deora et al. demonstrated the rapid internalization of anti-tumor necrosis factor (TNF) Abs following their formation of a complex with transmembrane TNF (tmTNF) (153). This complex is initially routed into early endosomes and subsequently transported to lysosomes, where it undergoes further degradation. Jin et al. also compared the internalization of different variant of avelumab using pHrodo labeling (75). Moreover, Siegel et al. also labeled different therapeutic Abs with a pH-sensitive fluorophore specifically directed to their Fc glycosylation site and compared their internalization rate in moDCs (5). Results revealed a linear correlation between the cellular accumulation of these Abs and their subsequent presentation by MHC-II molecules. Förster resonance energy transfer (FRET) is another method that allows the evaluation of internalized Abs. It requires the conjugation of the Ab with both a fluorophore and a quencher dye. At the cell surface, the fluorophore is masked by the quencher, and once internalized and degraded, the physical separation of the fluorophore and quencher results in a fluorescent signal detected by flow cytometry (157). This method was used in different studies to compare many Abs’ internalization rate and correlate with their immunogenic potential (8, 158, 159). However, since the FRET construct binds to the Fc region, Fc-mediated endocytosis could not be assessed, despite it being a potentially significant uptake pathway for many Abs. Recently, a group developed a new format for assessing Ab’s internalization based on cell surface staining and an intracellular staining of cells with an anti-human IgG F(ab’)_2_ following a 24 hours incubation with unlabeled mAb studies tested different Abs and compared their internalization rate and their immunogenic potential through different assay ( (4, 7).These different tools used to evaluate therapeutic Ab’s internalization are summarized in **Table 1**. Many *in vitro* assays are available based on different techniques to evaluate the role of a specific receptor in mAb’s internalization. Jin et al. demonstrate the implication of FcγR in avelumab’s uptake by testing and comparing it to FcγR binding–deficient variants (75). On the other hand, Xue et al. showed that an anti-IL21 receptor internalization by DCs is slightly dependent on FcγR as blocking these receptors by an Fc block did not modify the cellular binding (155). Other studies were based on competition or blocking experiments to identify a specific receptor. For example, Aoyama et al. identified the implication of FcγRs in ADC aggregates internalization by analyzing the uptake inhibition by flow cytometry after blocking FcγRIIa (148). Another study demonstrated the MMR-dependent endocytosis of factor VIII by blocking mannose-sensitive receptors after preincubating DCs with mannan (97). Another alternative consists of analyzing uptake by cellular model that overexpresses a particular receptor. After incubation with wild type or CD206-expressing Chinese hamster ovary (CHO) cells, a receptor-specific targeting and uptake was shown for nanocarriers by flow cytometry and confocal microscopy (160). Anderson et al. showed that using the same technique principle, the specific uptake of a glycopeptide in DC-SIGN CHO (+) was achieved (161).

**Table 1:** Tools to evaluate the internalization of therapeutic antibodies and their limitations.

Internalization test	Internalization test		Limitations
Quantification of internalization by flow cytometry and evaluation of lysosomal degradation by confocal microscopy	Direct labelling		Challenges related to the labelling Amine-reactive labeling can modify lysine residues critical for antigen binding or Fc receptor interaction, potentially reducing binding affinity or altering effector functions Labelling could increase the risk of structural destabilization and aggregation Challenges in Discriminating Between Surface-Bound and Effectively Internalized Material, and Limitations in Intracellular Trafficking Analysis Discriminating between surface-bound and effectively internalized material is challenging, particularly when not using confocal microscopy. Furthermore, a lack of detailed information on intracellular trafficking pathways adds complexity for interpreting experimental data. Challenges in Comparing Antibodies with Different Degrees of Labeling Comparisons between antibodies can be unreliable if the degree of labeling is not standardized or equivalent.
	External and internal labelling with an anti-human IgG F(ab’)2 or F(ab’)2 anti-human IgG (H+L) conjugated to a fluorophore		Partial assessment Using anti-F(ab’)2 labeling (either recognizing the Fab or Fc region) for tracking antibody internalization may hinder the ability to fully assess Fc-mediated or target-specific interactions. This could lead to an incomplete understanding of Ab trafficking, processing, and interaction with both Fc receptors and target molecules, limiting insights into the Abs’s full internalization pathway.
Quantification of lysosomal degradation by flow cytometry	FRET		Binding to the Fc Portion Binding exclusively to the Fc portion prevents the evaluation of Fc/glycosylation-dependent endocytosis mechanisms. This limitation arises because interactions with C-type lectin receptors (CLRs) or Fcγ receptors (FcγRs) are abrogated.
	pH-rodo labelling	Dye reactive to amine	Degrees of Labeling Comparisons between antibodies can be unreliable if the degree of labeling is not standardized or equivalent
		Dye directed to heavy chain N-linked glycans	Degrees of Labeling Comparisons between antibodies can be unreliable if the degree of labeling is not standardized or equivalent Challenges to analyze unglycosylated antibodies

Ab, antibody; Fab, fragment antigen-binding region; FcR, Fc receptors; FRET, Förster resonance energy transfer.

## 7 Conclusion

**Figure 3:**
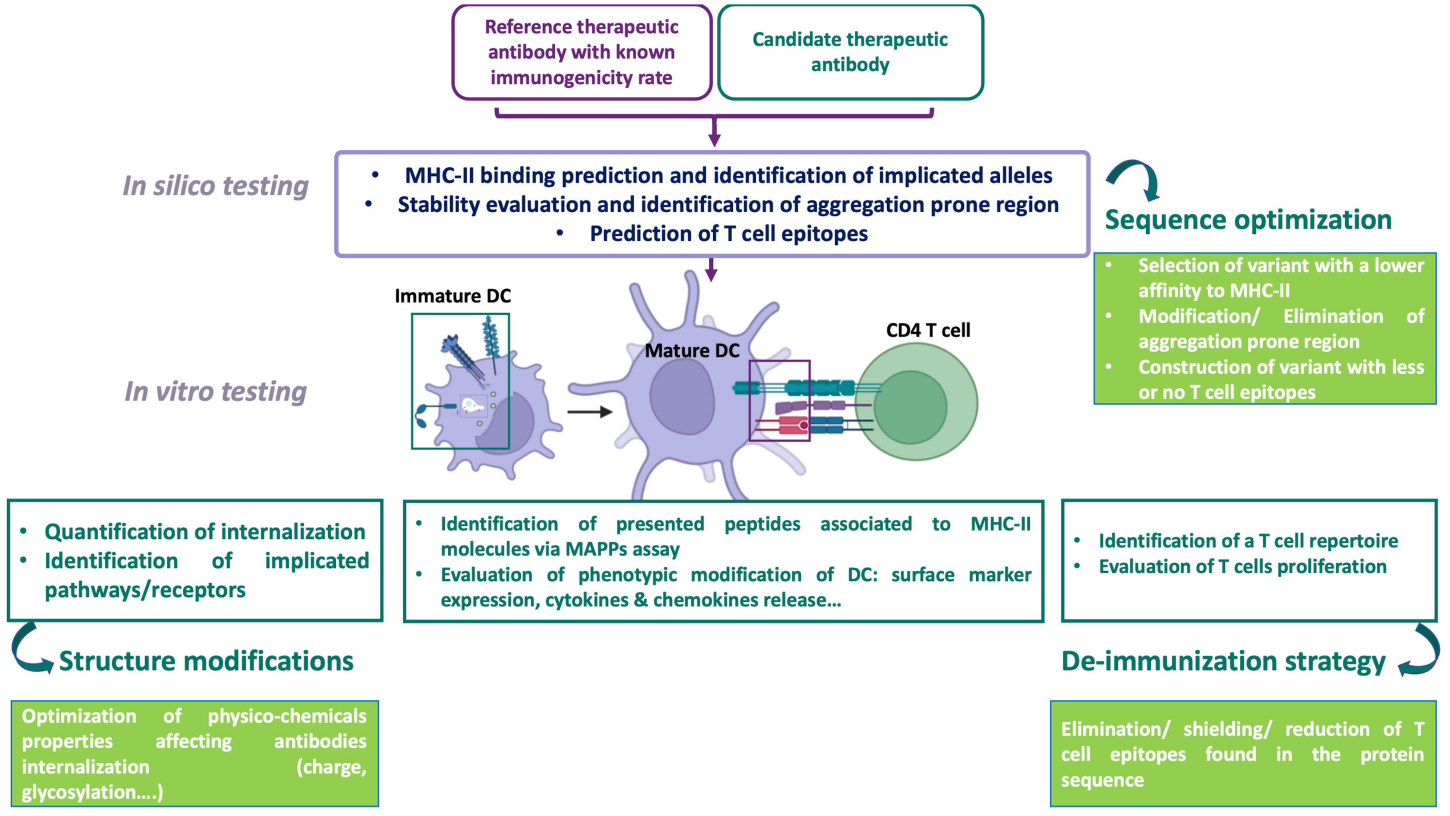
An evidence-based strategy for assessing the immunogenicity risk of therapeutic protein candidates, including an internalization assessment test. DC, dendritic cell; MAPPS, MHC-associated peptide proteomics; MHC, major histocompatibility complex, The immunogenicity of therapeutic antibodies is evaluated using complementary approaches. In silico evaluation, based on a database of therapeutic antibodies with known immunogenic risks, predicts MHC-II binding, identifies aggregation-prone regions (APRs), and assesses epitope-based risks and implicated alleles. This approach can guide antibody sequence optimization by selecting variants with lower MHC-II affinity, reduced APRs, and fewer T cell epitopes, while maintaining activity. The next step involves in vitro evaluation of the cellular mechanisms involved in T cell response initiation. Assessing the internalization and processing of antibodies by monocyte-derived dendritic cells (moDCs)—two key steps in this immune response—is important for quantifying antibody uptake and comparing it to a reference antibody with known immunogenicity. This is complemented by identifying internalization pathways and receptors using pharmacological inhibitors or receptor-overexpressing models, as antibody entry depends on properties such as glycosylation and charge. Optimization involves modifying the antibody’s physicochemical properties, such as testing different monoclonal antibodies (mAbs) with the same Fab but varying Fc portions (e.g., glycosylated versus non-glycosylated). The MAPPS assay complements internalization evaluation by identifying peptides presented on MHC-II molecules to CD4 T cells. Additionally, dendritic cell (DC) activation testing is crucial for assessing the biological activity of impurities or large aggregates. T cell assays encompass multiple stages of the immune response: internalization, antigen presentation, peptide recognition, and T cell activation. These assays either identify the presence of a T cell repertoire in response to an antibody, which is a prerequisite for T cell initiation, or assess T cell proliferation in response to the antibody. Based on these results, antibody sequence optimization may be considered to eliminate impurities and reduce immunogenicity by removing, masking, or reducing T cell epitopes. Creating using BioRender.com.

The development of an immune response against therapeutic Abs requires their internalization by APCs, mainly DCs, and their processing into peptides. Understanding how these Abs are internalized by cells and the influence of their physico-chemical properties on their uptake is critical for evaluating their immunogenicity. Here, we focused on Abs’ properties and the aggregation impact on their uptake by DCs, presented a general overview of the current understanding of endocytosis, and discussed current experimental techniques. Aspects of Abs’ properties, such as size, PTM, surface chemistry, and their aggregation propensity, are thought to influence the route and the rate of internalization. The current assays evaluating Ab’s internalization present some limitations, such as the evaluation of one main endocytic route, which might lead to false output. Considerable research effort is still expanding to develop tools to evaluate Ab’s internalization and integrate it into the overall evaluation of risk of immunogenicity. An increased understanding of these processes could greatly improve the ability to predict the risk of immunogenicity and facilitate the development of effective mitigation strategies. To this end, an evidence-based strategy is proposed for assessing the immunogenicity risk of therapeutic Abs candidates. This strategy includes internalization assessments, along with other evaluation methods that address various stages of the immune response. It also incorporates multiple risk-mitigation approaches, allowing for a more comprehensive and predictive evaluation of potential immunogenic reactions. Such a framework is essential for optimizing Ab design, minimizing the risk of adverse immune responses, and improving the overall safety and efficacy of Ab-based therapies (**Figure 3**).
